# A study to assess the efficacy of print and digital health communication media tools (HCMT) in rural and urban communities.

**DOI:** 10.12688/f1000research.139997.1

**Published:** 2023-10-11

**Authors:** Sushim Kanchan, Abhay Gaidhane

**Affiliations:** 1 School of Epidemiology and Public Health, Datta Meghe Institute of Higher Education and Research, Wardha, Maharashtra, 442001, India; 2 Community Medicine and School of Epidemiology and Public Health, Datta Meghe Institute of Higher Education and Research, Wardha, Maharashtra, 442001, India

**Keywords:** HCMT (Health communication media tool), print media, digital media, health information, health communication, mass media

## Abstract

**Background:** Existing studies have described the potential of either digital or print media for health information in one discipline. Both media are excellent tools for disseminating information, promoting social awareness, and offering in-depth healthcare information thereby being considered as leading health communication media tools. Hence, this study aims to assess and compare the effectiveness of print and digital media in various aspects of health communication in rural and urban populations.

**Methodology:** A cross-sectional population survey will be conducted in rural and urban areas, using a semi-structured, pre-tested questionnaire, which includes socio-demographic variables, media usage patterns, perception, and health behavior change from health information via both media. The study population size will include 342 individuals in the age group of 21 to 60 with minimum qualification of matriculation.

**Study implications:** Our research will help to understand which media are more effective at reaching different populations and can help choose appropriate communication channels for health promotion efforts, develop more effective interventions, and identify potential disparities in access to health information and resources.

## Introduction

Health communication via mass media (digital, electronic, and print) is an emerging field for health information, education, and promotion among communities. However, the advanced platforms of the Web 2.0 (internet platform) age have accelerated the battle against the print media, changing the nature of communication, including health-related communications. ^1^ Despite being more available and accessible health information channels, these modern digital media have discrete information, so somewhere the authenticity is being lost. On the other hand, print media believes to maintain health information’s reliability and credibility, ^2^ which is why the printing channels still exist. Similarly, electronic media (e-media) is an integral aspect of today’s era, and health care is no exception. ^3^ For example, the electronic health record in a healthcare organization, ^4^ mobile health (mhealth) contact tracing, and telemedicine are new faces of the health system. ^5^

Although it has also been apparent that not all of these media interventions for health-related reasons are successful. ^6^ For instance, sources may be effective in increasing patient awareness, but persistent use of print or digital media did not seem to persuade doctors, ^1^ and the problem of self-expertise has gotten worse. ^7^ Concerns also exist regarding news reports that are sufficiently erroneous or deceptive due to poor reporting or complete falsification. ^8^ Therefore, efficient use and the role of health communication media tools must be understood and further explored. Our study aims to know how capable these media (print and digital) are for health communication in various aspects.

### Rationale

The study will compare and analyze the performance of print and digital health communication media in both rural and urban communities, considering a range of relevant aspects like user engagement, beliefs, and behavioral change to gain comprehensive insights into their efficacy across sociodemographic variables. Understanding the capabilities of media tools in specific contexts can be helpful in the selection and utilization of appropriate media channels to maximize the reach and impact of health messages, bridge the gap in health communication and contribute to reducing health disparities and developing tailored communication strategies.

### Aim

The purpose of this research paper is to assess the effectiveness of print and digital media as health communication tools in urban and rural communities.

### Objectives

a) Primary objective:
  i. To assess the usage of print and digital health communication media tools among individuals in rural and urban communities.
  ii. To compare the perceptions of individuals in rural and urban communities for print and digital health communication media tools.
  iii. To explore the impact of print and digital health communication media tools on health behavior changes among individuals in rural and urban communities.
b) Secondary objective:
  i. To determine the association between population type and their consumption pattern, beliefs, and health behavior in response to health information acquired through the media.

## Methods

### Study design

A community-based cross-sectional study will be conducted during a 6-month survey from March 2023 to November 2023. A face-to-face interview will be conducted using a semi-structured, pre-tested questionnaire regarding how various media are used for health-related reasons, their reliability and accessibility, and changes in rural and urban populations’ health behavior using the mobile app ONA data kit. A minimum of 6-7 visits will be required to collect all the data, with each subject receiving a 10-minute survey and sign a consent form.

### Study settings

The current study will be conducted in the field practice area of the community medicine department under the Datta Meghe Institute of Higher Education and Research (DMIHER) for the rural population and urban population in the Wardha district.

### Participants

The study participants will be a population of age 21- to 60-year-olds with minimum qualification of matriculation in urban and rural areas of Wardha **.**

### Eligibility criteria

Individuals who have agreed to engage in the study will make up the study’s participants. A person will be excluded if they have a history of neuropsychiatric disorders, are physically challenged, have less education than a high school graduate (matric), and refuse to give their consent. After the initial visit, if a particular property is found to be locked, and after two additional attempts to contact the eligible residents, they will be eliminated from the study.

### Data sources

**Variables**

*Dependent variables*

Efficacy of health communication media tools (print and digital)

*Independent variables*

• Sociodemographic variable (age, sex, education, and population type: rural/urban,)
• Type of media preferred (print media, digital media)
• Frequency of media consumption (daily, weekly, monthly, rarely, never)
• Perception of accessibility and trustworthiness of print media for health-related information
• Perception of accessibility and trustworthiness of digital media for health-related information
• Influence of print media and digital media on health behavior

### Data collection tools and procedure

Data tool (Ona.io): Validated structured questionnaire will be created in ona.io (Odk- questionnaire).

To ascertain the effectiveness of print and digital health communication tools on members of rural and urban communities, the tool consists of a structured interview questionnaire that is semi-structured and close-ended. We modified a previously validated questionnaire ^6^^,^^9^^–^^11^ and prepared self-made questionnaire to compile both- print and digital media users about their viewpoints on capabilities of providing health information via both media as communication tool. The second author, a public health expert, evaluated and approved the final questionnaires.

The questionnaire consists of 17 multiple-choice questions divided over 4 sections ( Table 2) ^2^: Sociodemographic information, ^1^ Media Usage/Media consumption habit, ^3^ Perceptions of Health Communication Media Tools and ^4^ Effect of Health Communication Media Tools on Health Behavior. The questionnaire will include questions in English. For Marathi regional language speakers, a translator will be present during survey time for explaining questions to participants. A pilot study with a sample size of 33 participants was completed using the final set of questionnaires, and the full sample size was calculated because the standard data for sample size computation for both print and digital media together, were not readily available.

Data sources are presented in Table 1.

**Table 1.** Data sources.

Key study parameters	Variables	Data collection method	Data sources
**(Section A)** Sociodemographic profile	1. Age 2. Sex 3. Education 4. Population type: Urban/Rural	Survey using a Questionnaire	The field practice area of the community medicine department under the Dutta Meghe Institute of Higher Education and Research (DMIHER) for the rural population and urban population in the Wardha district.
**(Section B)** Media Usage/Media consumption habit	1. The type of media being preferred for health information. 2. Frequency of media consumption for health information.	Survey using a Questionnaire	
**(Section C)** Perceptions of Health Communication Media Tools	1. The accessibility and 2. credibility of health information in print and digital media.	Survey using a Questionnaire	
**(Section D)** Effect of Health Communication Media Tools on Health Behavior	1. The effect of health information acquired through various media outlets on health behavior like a. influence decision-making, b. engagement in preventive measures and c. information-sharing tendencies.	Survey using a Questionnaire	

**Table 2.** Questionnaires format/section.

**Section A:** Demographic variable	A semi-structured questionnaire that will be pre-tested, will be used to bring out independent variables like rural or urban, age, sex, and education.
**Section B:** Media Usage/Media consumption habit	The questionnaire will include questions about the respondents’ views on the types of media (print and digital) they utilize and the regularity with which they consult the media for health information.
**Section C:** Perceptions of Health Communication Media Tools	The questionnaire will ask about the accessibility and credibility/trustworthiness of health information in print and digital media. The question’s response five-point Likert scale ranging from ‘strongly agree to strongly disagree’.
**Section D:** Effect of Health Communication Media Tools on Health Behavior	The questionnaire will inquire about the influence of these media tools on health-related decision-making and information-sharing tendencies. Five-point Likert scale ranging from ‘strongly agree to strongly disagree’ and ‘very likely to not likely at all’ will be used.

### Data analysis plan

The study will be conducted using simple random sampling. A list of all rural areas under the field practice area of the community medicine department under the Datta Meghe Institute of Higher Education and Research (DMIHER) will be prepared and will be numbered using a random number generator, created in an Excel spreadsheet using the formula =RAND (). Once the list will be randomly sorted, the first two or three rural areas will be selected. The same process of selection will be performed for urban areas. The data will be entered into MS Excel, and descriptive statistics, including frequency and percentages, will be presented using a table and graph. Chi-square test will be performed to determine the association between category variables.

### Bias

Recall bias: Participants may have difficulty in accurately recalling their exposure to print and digital health communication media tools or their health-related behaviors. Shorter recall periods will be used to address the bias, meaning that instead of asking participants to recollect events over a long period of time, researchers can utilize shorter recall periods to reduce memory errors.

Response bias: Participants’ responses to the questionnaire may be influenced by social desirability bias, where they provide answers, they believe are socially acceptable or expected. In order to counteract this bias, an anonymity and secrecy approach will be used. Through this method, we can guarantee participants’ anonymity and confidentiality and provide a secure setting in which they can respond honestly without worrying about criticism or social ramifications.

Selection bias: There could be a risk of selection bias if the participants in the study are not representative of the larger population in rural and urban communities. Random sampling techniques will be used to help eliminate selection bias by guaranteeing that every member of the population has an equal chance of being included in the study.

### Study size

The sample size was decided after calculating the pilot study on 33 samples.

The estimated proportion with absolute precision: $n\geq \frac{Z^{2}1-\alpha /2\times p\left(1-p\right)}{d^{2}}$

Alpha (α): 0.05

Estimation error (p): 0.666

Estimation error (d): 0.05

The minimum sample size needed: 342

### Statistical method

Data will be entered into MS Excel software and analyzed using SPSS version 22 (RRID:SCR_002865). The descriptive statistics (frequencies and percentages) for sociodemographic information, popular media, frequency of media consumption, accessibility, credibility, influence of media in health-related decisions, and information-sharing tendencies of health information related data will be used. Chi-square will be employed to measure the association between sociodemographic variables and their choice of health communication media tool.

**Expected outcomes**

*Media preference:* The study may reveal that digital media tools are more frequently used and preferred by individuals in both rural and urban communities compared to print media tools.

*Reach and accessibility:* The research may demonstrate that digital media tools have a broader reach and higher accessibility compared to print media tools in both rural and urban communities. Digital platforms may provide a more convenient and readily available source of health information for individuals in diverse locations.

*Perceived credibility*: The study may reveal that print media are more trustworthy for health information as compared to dispersed digital information.

*Influence on decision-making:* The study could reveal that both print and digital media tools have a significant influence on individuals’ health-related decision-making processes in a positive way.

*Behavior change:* The findings may indicate that individuals exposed to print and digital media tools are more likely to engage in positive health behaviors like adopting healthier lifestyles, seeking preventive healthcare measures, or adhering to recommended treatment plans.

*Demographic differences:* The findings may reveal that younger individuals and those with higher educational backgrounds are more likely to utilize digital media tools and exhibit greater behavior changes compared to older individuals and those with lower educational backgrounds.

**Key results**

The findings can be used to determine the preferred media tool (print or digital) and levels of interaction of print and digital media among people in urban and rural locations, based on factors like accessibility and perceived legitimacy. This might entail ideas for combining print and digital media tools, tailoring messages for audiences, utilizing preferred media platforms, or removing obstacles to media access. Thus, the study can help policymakers, public health organizations, and healthcare practitioners establish policies and standards for successful health communication projects and use media channels cost-effectively.

**Limitations**

One limitation of the study is that it will not accurately provide information about the frequent health topics covered in the media and accuracy of these health information. People with qualifications below matriculation are omitted, thus their familiarity with health information is not examined. A further omission from the study is any discussion of the effectiveness of various types of print media (such as newspapers, posters, books, etc.) and digital media (such as social media and the internet) in rural and urban populations.

**Dissemination**

The results will be published in relevant indexed journals.

**Study status**

The piloting of the data collection tool was done. Recruitment of final study participants is scheduled in the coming months.

## Discussion

It has been proposed that the use of social networking sites to share credible health information can assist physicians in fulfilling their professional obligation to transmit pertinent information to patients, colleagues, and the public, as well as assist members of the public in properly contextualizing the findings of health-related current events. ^12^ Similar studies by Belt *et al.* in 2013 found that one in four people would interact with their doctor through social media, and it is anticipated that this figure will rise. As a result, healthcare professionals ought to investigate new Internet communication channels and encourage patient interaction. ^6^

The study conducted by Fatimah *et al.* in North Toraja society in 2021 shares a perspective on the choice of printed communication media, such as books and magazines, as a source of knowledge on reproductive health. He calculated that print media garners 57.39% of preferences, followed by websites (27.8%) and social media (14.78%). ^13^ Online medical education can be an effective method for medical students ^14^ and an informative and communicative data source for health researchers. ^15^

The public’s perceptions of health-related concerns can be influenced by the mass media by stressing specific aspects of its coverage, such as the origins of issues, the availability of solutions, as well as post-disease treatment, and precautions. According to a 2021 study by Ghosh *et al.* on how reversible current procedures give women greater contraceptive options than opting for permanent therapies that restrict their capacity to bear children, the media effectively emphasizes and raises awareness of voluntary contraceptive use in India. The decomposition analysis’s findings revealed that media exposure boosted the utilization of reversible advanced treatments by 14%. ^16^ The digital tool has the potential to increase the frequency with which sexual health is discussed in primary care, notably by nurses, leading to an improvement in patient satisfaction, 2010 research by Macdowall *et al.* ^17^ Similarly, the results and analysis of the survey conducted by Pandey and Kumar in 2020 indicate that print media has been crucial in raising awareness of the pandemic Coronavirus Disease (COVID-19), supporting the idea that the media may be a useful tool in times of national emergency. ^18^

Based on the findings of these previous studies, our planned study seeks to expand the understanding of the role of communication media in the healthcare context by focusing on the efficacy and impact of both print and digital health communication media in rural and urban communities. While the existing studies have shed light on various aspects of media’s influence on health-related information dissemination and public perception, our study aims to contribute by directly comparing the performance of print and digital media across diverse population types, thereby offering valuable insights into enhancing health communication strategies.

### Ethical consideration

With the written consent of the head of the relevant institution, data collection will happen at a time that is convenient for instructors and rural and urban community members. The institutional ethics committee of the Datta Meghe Institute of Higher Education and Research (DMIHER) has approved the research proposal with the approval number DMIHER (DU)/IEC/2023/623. Before beginning our study, we will also obtain written informed consent and notify the participants of its purpose. We will make sure the interviewee has privacy and feels comfortable throughout the interview.

## Data Availability

No data is associated with this study as this is a study protocol. Zenodo: A study to assess the efficacy of print and digital health communication media tools (HCMT) in rural and urban communities, https://doi.org/10.5281/zenodo.8238040. ^19^ This project contains the following extended data:
- Questionnaire Data are available under the terms of the Creative Commons Attribution 4.0 International license (CC-BY 4.0).
