# Supplementary material for: A study to assess the efficacy of print and digital health communication media tools (HCMT) in rural and urban communities
Source: F1000Res. 2023 Oct 11;12:1314. [Version 1] doi: 10.12688/f1000research.139997.1 (PMC11459119; doi:10.12688/f1000research.139997.1)
Supplement: Peer review history

# Reviewer report: Reviewer response for version 1

**DOI:** 10.5256/f1000research.153324.r311654

**Published:** 2024-10-07

**Authors:** Debdutta Bhattacharya

**Affiliations:** 1 ICMR - Regional Medical Research Centre, Bhubaneswar, India

The study protocol presents a valuable investigation into the effectiveness of health communication media in rural and urban communities. However, it is essential to provide detailed information about the Mobile App ONA data kit used for data collection, including its reliability, functionality, and user experience, to enhance the transparency and credibility of the research methodology.

Is the study design appropriate for the research question?

Yes

Is the rationale for, and objectives of, the study clearly described?

Yes

Are sufficient details of the methods provided to allow replication by others?

Yes

Are the datasets clearly presented in a useable and accessible format?

Yes

Reviewer Expertise:

Health Communication, One Health, AMR, Microbiology, Public Health

I confirm that I have read this submission and believe that I have an appropriate level of expertise to confirm that it is of an acceptable scientific standard.

# Reviewer report: Reviewer response for version 1

**DOI:** 10.5256/f1000research.153324.r321994

**Published:** 2024-10-03

**Authors:** Kavita Thapliyal, Manjul Thapliyal

**Affiliations:** 1 Amity University, Noida, Uttar Pradesh, India; 2 CSR, Public Health & IT, Principal Consultant- Visions Ahead Foundation, Noida, Uttar Pradesh, India

Merits:

 The well written article is aptly relevant to the study of disseminating public health information through print and digital media platforms. Primary and Secondary objectives are also well defined. Methodology of cross sectional population design is also appropriate with a semi structured and pre tested questionnaire making it flexible for qualitative inputs while having a quantitative approach. The age group, qualification and demographics provide a uniform base for desired output, with Department of DMIHER further validating the study. 

 Limitations & Scope of Improvement : 

 I feel a Longitudinal design would have given better output against a cross sectional design giving accurate behavior changes. The area of study has limited scope of study in lieu of minimum qualification, general accessibility, self reporting bias, sample size justification, data collection, timeframe with digital platform and net availability. Add more relevant referencing to make it a enriching read.

Is the study design appropriate for the research question?

Yes

Is the rationale for, and objectives of, the study clearly described?

Yes

Are sufficient details of the methods provided to allow replication by others?

Yes

Are the datasets clearly presented in a useable and accessible format?

Yes

Reviewer Expertise:

Digital Twin, Chatbot XAI, AI & Neurodegenerative Treatment, Social Media & Health Communication, Cyber Phycology, GHRM, Cross Culture Management

We confirm that we have read this submission and believe that we have an appropriate level of expertise to confirm that it is of an acceptable scientific standard.

## References

[1] : Improving health literacy using the power of digital communications to achieve better health outcomes for patients and practitioners. *Front Digit Health* .2023;5: 10.3389/fdgth.2023.1264780 1264780 10.3389/fdgth.2023.1264780 38046643 PMC10693297

[2] : Demand of and Access to Health Messages Through Mass Media in the Rural Community of Eastern Ethiopia: A Mixed Method Study. *Risk Manag Healthc Policy* .2023;16: 10.2147/RMHP.S429712 1859-1874 10.2147/RMHP.S429712 37719689 PMC10503334
